# Mt. Sinai Hospital Reports

**Published:** 1899-10

**Authors:** 


MT. SINAI HOSPITAL REPORTS. Volume I. 1898. Edited for the medical board by PAUL F. MUNDÉ, M. D., L.L. D. 1899.

Although Mount Sinai Hospital has existed since 1856 and the number of patients have increased annually from 200 to 3,000, the vast material has been thus far insufficiently utilised in the interest of medical science and art. Indeed very few publications based upon the experience gathered in the hospital have seen the light. At last the board of directors have granted the means for publishing an annual volume and henceforth the staff at Mount Sinai Hospital, under the able leadership of Dr. Abraham Jacobi, will be able to collate their experiences and investigations in a manner alike satisfactory and commendable. Some idea of the work done by this hospital can be gleaned by the statistics of the medical and surgical services during the year 1898.

In the medical wards there were treated 1,020 patients, of whom 494 were cured, 351 improved, 54 unimproved, and 121 died. Among the rare diseases seen here mention may be made of cases each of acromegaly, Landry's paralysis, syringomyelia and astasia abasia. Among some of the contributions of this volume mention may be made of the following. A study of the cases of typhoid fever observed in the hospital, 1883-1898, by J. Rudisch; Statistics of 500 cases of lobar pneumonia, by Alfred Meyer; Notes on some interesting results with the Widal test, by Alfred Meyer; A case of pernicious anemia with fatty heart occurring during pregnancy, by Alfred Meyer; A case of abscess of the liver which ruptured into the lung, by N. E. Brill; A case of ulcerative endocarditis involving the valve of the pulmonary artery, by N. E. Brill; Acute pancreatitis—disseminated fat necrosis of omentum and peritoneum—laparatomy—recovery, by Morris Manges; Multiple carcinosis of bones secondary to carcinoma of breast, Morris Manges; Study of a case of echinoccocus cyst of the liver with discharge of daughter cysts through the common bile duct, by Henry W. Berg; Statistics from the children's department, by Barnim Scharlau; On erythromelalgia, by B. Sachs; report of the department of general surgery, by Arpad G. Gerster; Constricting adenoma of the hepatic flexure—recovery after extensive resection of the colon, by Howard Lilienthal; Statistics of the genito-urinary department, by William F. Fluhrer; three cases of prostatectomy, by William F. Fluhrer; gynecological service, by Paul F. Mundé; Vaginal celiotomy for diseases of the appendages, by J. Brettlauer; Statistics of ear and eye service, by E. Gruening; The mastoid operation in acute empyema and caries, by E. Gruening; Restoration of the conjunctival cul-de-sac in a case of total symblepharon by means of Thiersch skin grafts, by Charles H. May; Report by Carl Koller; A case of thrombo-phlebitis of the sigmoid sinus and the jugular vein, by Carl Koller; A report of a series of unusual pathologic conditions, by F. S. Mandlebaum and E. Libman; A review of the Widal tests made during 1898, with a description of the method used, by E. Libman.

These reports will take rank with the reports of the Boston General, Johns Hopkins, Brooklyn Methodist Episcopal and New York Presbyterian Hospitals, and should act as an incentive for other hospitals to do likewise. Dr. Jacobi and his staff are to be heartily congratulated. W. C. K.

